# Compositional alterations of gut microbiota in nonalcoholic fatty liver disease patients: a systematic review and Meta-analysis

**DOI:** 10.1186/s12944-021-01440-w

**Published:** 2021-02-26

**Authors:** Fuxi Li, Junzhao Ye, Congxiang Shao, Bihui Zhong

**Affiliations:** Department of Gastroenterology, The First Affiliated Hospital, Sun Yat-sen University, NO 58 Zhongshan II Road, Yuexiu District, Guangzhou, 510080 P. R. China

**Keywords:** Nonalcoholic fatty liver disease, Nonalcoholic steatohepatitis, Gut, Microbiome, Bacterial composition, Microbiota

## Abstract

**Background:**

Although imbalanced intestinal flora contributes to the pathogenesis of nonalcoholic fatty liver disease (NAFLD), conflicting results have been obtained for patient-derived microbiome composition analyses. A meta-analysis was performed to summarize the characteristics of intestinal microbiota at the species level in NAFLD patients.

**Methods:**

Following the Preferred Reporting Items for Systematic Reviews and Meta-Analyses (PRISMA) Statement, a completed search (last update: December 30, 2020) of databases was performed to identify eligible case-control studies detecting gut microbiota in NAFLD patients. The meta-analysis results are presented as the standard mean difference (SMD) and 95% confidence interval (CI). Bias controls were evaluated with the Newcastle-Ottawa Scale (NOS), funnel plot analysis, and Egger’s and Begg’s tests.

**Results:**

Fifteen studies (NOS score range: 6–8) that detected the gut microbiota in the stools of 1265 individuals (577 NAFLD patients and 688 controls) were included. It was found that *Escherichia*, *Prevotella* and *Streptococcus* (SMD = 1.55 [95% CI: 0.57, 2.54], 1.89 [95% CI: 0.02, 3.76] and 1.33 [95% CI: 0.62, 2.05], respectively) exhibited increased abundance while *Coprococcus*, *Faecalibacterium* and *Ruminococcus* (SMD = − 1.75 [95% CI: − 3.13, − 0.37], − 9.84 [95% CI: − 13.21, − 6.47] and − 1.84 [95% CI, − 2.41, − 1.27], respectively) exhibited decreased abundance in the NAFLD patients compared with healthy controls. No differences in the abundance of *Bacteroides*, *Bifidobacterium*, *Blautia*, *Clostridium*, *Dorea*, *Lactobacillus*, *Parabacteroides* or *Roseburia* were confirmed between the NAFLD patients and healthy controls.

**Conclusions:**

This meta-analysis revealed that changes in the abundance of *Escherichia*, *Prevotella*, *Streptococcus*, *Coprococcus*, *Faecalibacterium* and *Ruminococcus* were the universal intestinal bacterial signature of NAFLD.

**Supplementary Information:**

The online version contains supplementary material available at 10.1186/s12944-021-01440-w.

## Background

Nonalcoholic fatty liver disease (NAFLD) is characterized by excessive intrahepatic lipid accumulation and consequent necroinflammation and fibrosis under conditions of metabolic disturbance, and it represents the leading cause of chronic liver disease worldwide [1]. The global prevalence of NAFLD is greater than 25%, and these levels are dramatically increased in areas with previously low incidence rates, especially in China [2]. Although the pathogenesis of NAFLD remains unclear, interactions between the environment and individual genetic backgrounds promote disease susceptibility. Moreover, the gut microbiota, which are shaped by the host immune response, and environmental factors, such as a high caloric diet, dietary fiber deficiency and a sedentary lifestyle, contribute greatly to the predisposition to NAFLD [3].

In clinical studies, ample evidence suggests that aberrations in the gut microbiota may play a key role in the development of NAFLD. Small intestinal bacterial overgrowth is more prevalent in NAFLD patients than in healthy individuals [4, 5]. High levels of lipopolysaccharide (LPS), the major constituent of the cytoplasm of gram-negative bacteria, were identified as an independent risk factor for NAFLD incidence [6]. *Escherichia* enriched in NAFLD patients is capable of ethanol synthesis [7], inducing oxidative stress that is involved in NAFLD progression. Changes in the community structure of intestinal microbes are also associated with inflammatory activity and the fibrosis stage of NAFLD [8]. Moreover, modulation of the gut microbiota by probiotic treatment has been demonstrated to improve liver injuries, metabolic abnormalities, and inflammatory chemokine levels in NAFLD patients [9]. Therefore, identifying the microflora signature in NAFLD is of great value because imbalanced microbiota may serve as a novel target for establishing diagnostic or treatment methods.

Although the gut microbiota is profoundly important in the pathogenesis of NAFLD, the distinct composition of the gut microbiota in NAFLD patients remains under debate. A limited number of studies on stool microbiota differences between NAFLD patients and controls have reported inconsistent or even opposing results. These discrepancies may be associated with geographic origins and dietary habits [10]. Therefore, we performed a meta-analysis to explore the profiles of intestinal dysbiosis in NAFLD patients in regions around the world.

## Methods

### Data sources

This systematic review and meta-analysis is reported following the Preferred Reporting Items for Systematic Reviews and Meta-Analyses (PRISMA) Statement [11] and the PRISMA 2009 checklist, which has been with a registration code in PROSPERO registry (registration number is: CRD42020220632, https://www.crd.york.ac.uk/PROSPERO). A complete and computerized search (last update: December 30, 2020) was performed without restriction based on region, language or publication type in the following electronic databases: PubMed, EMBASE and Cochrane Library. The Medical Subject Heading (MeSH) database was used to acquire MeSH terms and their relevant entry terms based on the following formatted terms: (NAFLD OR NASH OR NAFL OR “nonalcoholic fatty liver disease” OR “nonalcoholic steatohepatitis” OR “nonalcoholic fatty liver disease” OR “nonalcoholic steatohepatitis” OR “fatty liver” OR “steatohepatitis” OR “steatosis”) AND (microbiota OR microbes OR microbiome OR microflora OR flora OR bacteria). The query was adapted to different databases with minimal differences as required. The literature search was also supplemented with a manual search using the Related Articles Function and the reference lists of all retrieved articles.

### Study selection

Any study that provides necessary data on detecting gut microbiota for NAFLD patients and comparable controls was eligible for this systematic review and meta-analysis. The inclusion criteria for this study were as follows: 1) original full-text publications, 2) NAFLD diagnosed with sonographic or histologic evidence, 3) healthy controls with comparable age and sex proportions as the case group, 4) studies comparing the gut microbiota between NAFLD patients and healthy controls, and 5) available and sufficient data (sample size, mean and standard deviation or any data that can be converted to) to calculate the standardized mean difference (SMD) with 95% confidence interval (CI) in these two groups.

The exclusion criteria were as follows: 1) editorials, letters to the editor, reviews, case reports, hypotheses, book chapters, conference abstracts or studies on animals or cell lines; 2) studies that included NAFLD patients with other liver diseases, such as alcoholic fatty liver disease, autoimmune liver disease and viral hepatitis; and 3) studies that performed any intervention on participants at baseline.

### Data extraction

Two investigators (FX L and JZ Y) were responsible for selecting studies and extracting data from the included studies independently according to predesigned inclusion and exclusion criteria. Any disagreement was discussed and finally resolved by the adjudicating senior author (BH Z). If additional data or data transformations were required for analysis, the corresponding or first authors were contacted by email for assistance. When the authors did not reply, standard statistical formulas were applied for data transformation [12, 13]. If important additional data were not available from the corresponding or first author, the study was excluded.

A full-text review of the included studies was performed independently by the two aforementioned investigators, and the following variables were collected: 1) first author; 2) year of publication; 3) location; 4) study design; 5) number, age and sex of NAFLD (or NAFL or NASH) patients and healthy controls; 6) methods for diagnosing NAFLD; 7) methods for detecting and analyzing gut microbiota; and 8) bacterial counts and units for expressing the values.

### Quality assessment

To evaluate the risk of bias of individual studies, the methodological quality of the included studies was assessed using the Newcastle-Ottawa Scale (NOS) by two investigators (FX L and CX S). A senior author (BH Z) who was not involved in the initial assessment was responsible for adjudication on any disagreement between the two investigators. The NOS is a widely and frequently used tool for assessing the quality of nonrandomized studies included in systematic reviews and/or meta-analyses. Using this tool, study quality is assessed based on eight items that are categorized into three groups: study group selection, group comparability (a maximum of two stars can be given for comparability), and exposure or outcome of interest determination for case-controlled or cohort studies. A star rating system was used to rate the quality of the included studies based on a scale ranging from 0 (low quality) to 9 (high quality).

### Statistical analysis

In this study, statistical analyses were performed using R language Version 3.4.3 (The package of meta and metafor) and STATA/SE 12.0 (StataCorp, Texas, USA) with a Meta module for Windows. After extraction and transformation, all bacterial count data are presented as the mean ($\overline{\mathrm{x}}$), standard deviation (SD) and total sample size (n). The standardized mean difference (SMD) with 95% confidence interval (CI) was adopted to evaluate the effect size when a bacterial genus was detected by different techniques in the included studies [14]. The α criterion was set at 0.05. Accordingly, when the total SMD of a bacterial genus was more than 0 (favors NAFLD) with a 95% CI not including the value 0, we assumed that this bacterial genus exhibited overgrowth in NAFLD patients. Otherwise, the bacterial genus was assumed to be deficient in NAFLD patients. Additionally, subgroup analysis was performed based on the areas where these studies were conducted (Western and Eastern). Meta regression analysis was also performed to evaluate the effect of alanine aminotransferase (ALT) and body mass index (BMI) levels on mean different abundance of bacterial genus for NAFLD versus control patients. Those univariate significant variances would be entered in the multivariate models.

Heterogeneity across the included studies was evaluated using χ^2^ and *I*^2^ statistics. Higher χ^2^ and *I*^2^ statistics indicated greater heterogeneity, and an *I*^2^ value greater than 50% with a *P*-value less than 0.1 represented substantial heterogeneity in this analysis. A random-effects model was reported when substantial heterogeneity existed. The 95% prediction intervals which reflected the true effects of future similar studies with 95% certainties were calculated based on the formulas reported by Joanna IntHout et al. [15].

To assess the risk of publication bias, funnel plot analyses, Egger’s test and Begg’s test were conducted for genus-specific gut microbiota. Publication bias can cause asymmetry in funnel plots, which can be quantified with Egger’s and Begg’s tests. When *P*-values derived from Egger’s or Begg’s regressions were greater than 0.05, we assumed that no publication bias was present. Moreover, a sensitivity analysis was performed to evaluate the impact of each study on the overall results. In addition, considering the observational design limitations of each included study despite of its large sample size and quality, there would be the maximum certainty of an effect of case-control comparison, which was defined as credibility ceiling. Credibility ceilings tests setting different degrees of credibility to further adjust the included study in a meta-analysis would favor lessening the reporting of exaggerated associations with statistic significant results, therefore decrease the unmeasured bias. A sensitivity analysis based on ceiling value(s) of 0, 20 and 40% were conducted for ccredibility ceilings tests as the literature reported [16].

## Results

### Study selection

**Fig. 1:**
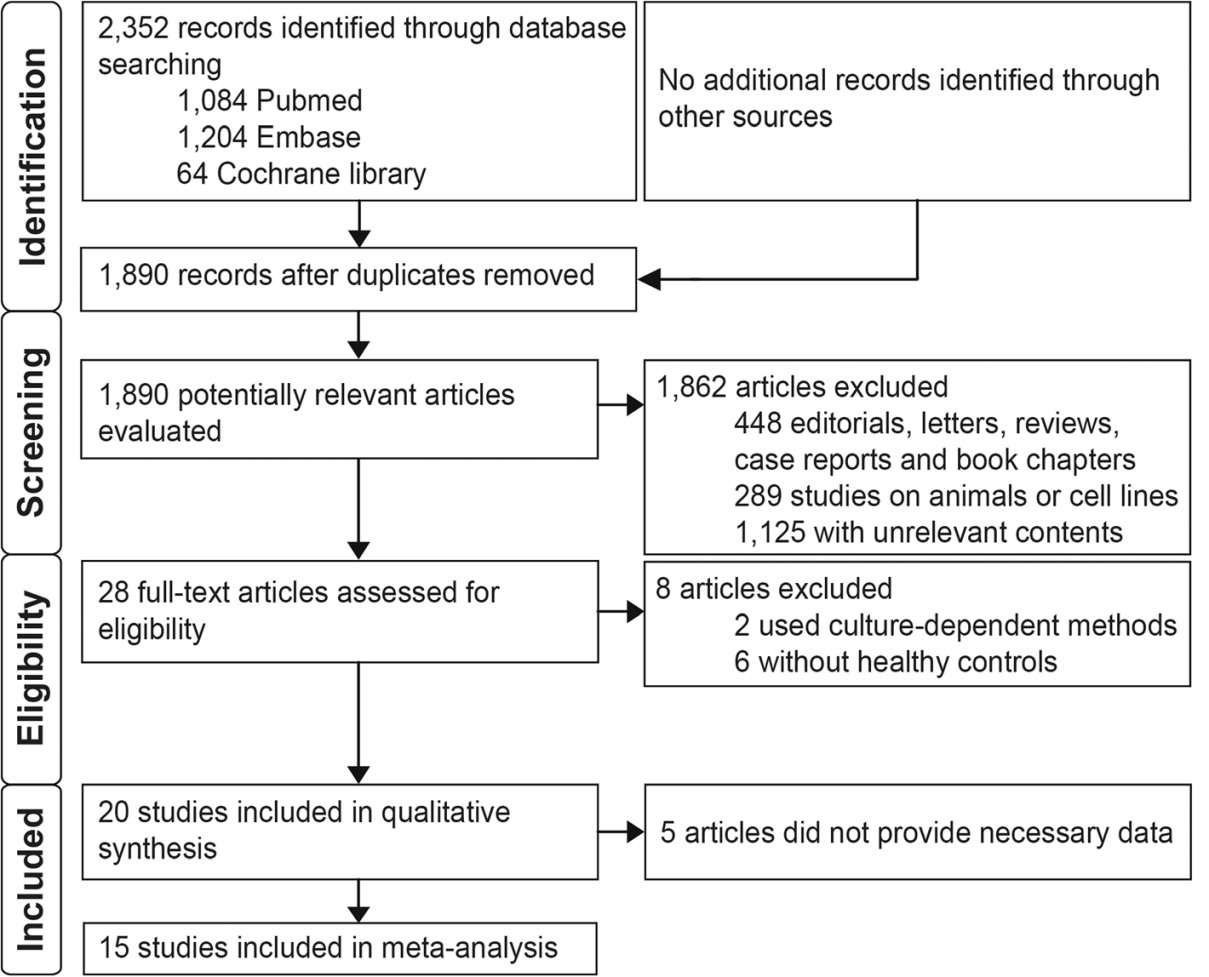
PRISMA flow diagram of studies identified in the meta-analysis. Abbreviations: PRISMA, Preferred Reporting Items for Systematic Reviews and Meta-Analyses

Using the predefined search strategy, 2352 citations were identified, as shown in Fig. 1. No additional records were identified through other sources. After removing duplicates, 1890 citations were screened by reviewing titles and abstracts. Of the remaining citations, 1862 articles were excluded because of unsuitable article type or studies on animals or cell lines or irrelevance. Then, the full texts of the remaining 28 articles were retrieved and assessed for eligibility. Of these full-text studies, 2 were excluded for using culture-dependent methods for detecting gut microbiota, 6 were excluded for not including a control group, and 5 were excluded because the authors did not provide necessary data. Finally, 15 studies published from 2012 to 2020 were included in the meta-analysis [7, 17–30]. Additional examination of the references listed in these studies did not provide any additional eligible studies. Agreement between the two investigators on study selection was 93.3%.

### Characteristics of the included studies

The main characteristics of the 15 included studies are listed in Table 1. Among the 15 studies consisting of 1265 individuals (577 NAFLD patients and 688 controls), six studies were performed in China, including one in Taiwan, one in Hong Kong, two in the USA, two in Canada, one in Thailand, one in Turkey, one in Spain, one in Italy and one in Korea. Nine of the 15 included studies adopted liver biopsy to diagnose NAFLD. All of the studies collected stools of the patients and healthy controls as samples for gut microbiota detection and utilized 16S rRNA sequencing.

**Table 1:** Characteristics of the included studies in the meta-analysis

First Author	Year	Location	Diagnostic methods	Numbers of NAFLD/Control	Age, years		Sex (Males, %)		BMI, kg/m^**2**^		ALT, U/L		Sample	Microbiota detection method
					NAFLD Control		NAFLD	Control	NAFLD	Control	NAFLD	Control		
Caussy [17]	2019	USA	Histology	42/51	NAFL54.0 ± 14.9NASH65.1 ± 9.8	45.9 ± 19.9	13 (31.0%)	15 (29.4%)	NAFL31.1 ± 6.6NASH31.3 ± 6.1	26.2 ± 6.8	NAFL23.2 ± 11.2NASH45.0 ± 37.4	18.8 ± 8.8	Stool	Sequencing
Chierico [18]	2016	Italy	Not mentioned	53/62	NAFL 12.04 ± 2.8NASH 12.27 ± 2.5	Healthy 10.24 ± 2.5Obese 11.25 ± 2.7	32 (60.4%)	26 (41.9%)	NAFL26.46 ± 4.43NASH27.42 ± 6.45	Healthy 17.59 ± 1.79Obese26.15 ± 4.38	NAFL32.3 ± 22.74NASH44.46 ± 16.73	HealthyNAObese 41.50 ± 47.70	Stool	Sequencing
Jiang [19]	2015	China	Histology or ultrasound	53/32	48.00	41.00	26 (49.1%)	5 (15.6%)	26.4 (21.5–33.3)	22.5 (18.2–33.5)	42.7 (11–145)	21 (6–29)	Stool	Sequencing
Li [20]	2018	China	Ultrasound	30/37	47.53 ± 8.5	44.24 ± 9.2	15 (50%)	11 (29.7%)	27.19 ± 2.56	23.37 ± 2.21	27.0 ± 17.63	16.7 ± 8.51	Stool	Sequencing
Nistal [21]	2019	Spain	Ultrasound	36/17	49.70 (31–64)	40.12 (25–56)	14 (38.8%)	5 (29.4%)	45.64 (38.9–61.1)	46.9 (39–63)	34.22 (15–91)	27.12 (10–52)	Stool	Sequencing
Özkul [22]	2017	Turkey	Histology	46/38	48.0 ± 12.0	36.0 ± 10.0	22 (47.8%)	12 (31.5%)	29.0 ± 4.0	22.0 ± 2.0	50.0 ± 41.0	20.0 ± 11.0	Stool	Sequencing
Raman [23]	2013	Canada	Ultrasound	30/30	49 (34–57)	51 (57–56)	13 (43.3%)	13 (43.3%)	33 (29–35)	22 (21–24)	51 (31–82)	18 (14–23)	Stool	Sequencing
Shen [24]	2017	China	Histology	25/22	45.5 ± 10.1	50.5 ± 9.5	19 (76.0%)	17 (73.7%)	28.6 ± 3.5	21.6 ± 1.7	51.6 ± 34.5	17.7 ± 5.3	Stool	Sequencing
Silva [25]	2018	Canada	Histology	39/28	NAFL48 (33–61)NASH46.5 (29–68)	36.5 (21–58)	20 (51.3%)	15 (53.6%)	NAFL27.4 (23.5–44.2)NASH32.1 (24.17–49.53)	26.6 (19.5–35.3)	NAFL45(14–116)NASH70(22–168)	17.5 (7–41)	Stool	Sequencing
Sobhonslidsuk [26]	2018	Thailand	Histology	16/8	59.8 ± 9.6	43.4 ± 6.8	3 (18.8%)	0 (0%)	27.7 ± 4.8	21.3 ± 1.2	59 ± 30	17 ± 6	Stool	Sequencing
Tsai [27]	2020	China (Taiwan)	Histology	50/25	51.2 ± 15.0	36.7 ± 15.0	24 (48.0%)	12 (48.0%)	31.3 ± 8.9	24.8 ± 5.2	50.2 ± 43.1	22.5 ± 15.7	Stool	Sequencing
Wang [28]	2016	China	Ultrasound	43/83	47.0 ± 12.0	40.5 ± 16.0	36 (83.4%)	70 (84.3%)	23.19 (22.19–24.22)	21.77 (20.7–23.38)	29 (20.5–39.5)	14.5 (12–20.75)	Stool	Sequencing
Wong [29]	2013	China (HongKong)	Histology	16/22	51 ± 9	44 ± 10	9 (56.3%)	9 (40.9%)	29.1 ± 5.6	22.2 ± 2.7	80 (44–94)	22 (17–30)	Stool	Sequencing
Yun [30]	2019	Korea	Histology	76/192	45.3 ± 8.2	42.9 ± 8.2	55 (72.4%)	83 (43.2%)	25.7 ± 2.6	22.2 ± 2.4	24.5 ± 12.9	20.9 ± 17.9	Stool	Sequencing
Zhu [7]	2012	USA	Histology	22/41	13.6 ± 3.5	Healthy14.4 ± 1.8Obese12.7 ± 3.2	12 (54.5%)	23 (56.1%)	34.0 ± 0.4	Healthy20.4 ± 0.1Obese33.4 ± 0.3	66.9 ± 1.9	HelathyNAObese 27.7 ± 0.6	Stool	Sequencing

Rank by the beginning letter of the first authors. *Abbreviations*: *NAFLD* nonalcoholic fatty liver disease, *NAFL* nonalcoholic fatty liver, *NASH* nonalcoholic steatohepatitis, *BMI* body mass index, *ALT* alanine aminotransferase

### Quality assessment of the included studies

As shown in Table 2, quality assessment of the included studies was carefully performed using the NOS, and the general quality was moderate (six studies scored 8 points, seven scored 7 points, and two scored 6 points, mean ± SD: 7.27 ± 0.70). Agreement between the two investigators for quality assessment was 93.3%. No study was excluded due to poor NOS score.

**Table 2:** Quality assessment of included studies using the Newcastle-Ottawa Scale (Case-Control Studies)

First Author	Selection^a^				Comparability^b^		Exposure^c^			Score
	1	2	3	4			A	B	C	
Caussy [17]	☆	☆	☆	☆	–	☆	☆	☆	–	7
Chierico [18]	–	☆	☆	☆	–	☆	☆	☆	–	6
Jiang [19]	☆	☆	☆	☆	–	☆	☆	☆	–	7
Li [20]	☆	☆	☆	☆	☆	☆	☆	☆	–	8
Nistal [21]	☆	☆	☆	☆	–	☆	☆	☆	–	7
Özkul [22]	☆	☆	–	☆	–	☆	☆	☆	–	6
Raman [23]	☆	☆	☆	☆	☆	☆	☆	☆	–	8
Shen [24]	☆	☆	☆	☆	☆	☆	☆	☆	–	8
Silva [25]	☆	☆	☆	☆	–	☆	☆	☆	–	7
Sobhonslidsuk [26]	☆	☆	☆	☆	–	☆	☆	☆	–	7
Tsai [27]	☆	☆	☆	☆	–	☆	☆	☆	–	7
Wang [28]	☆	☆	☆	☆	☆	☆	☆	☆	–	8
Wong [29]	☆	☆	☆	☆	☆	☆	☆	☆	–	8
Yun [30]	☆	☆	☆	☆	–	☆	☆	☆	–	7
Zhu [7]	☆	☆	☆	☆	☆	☆	☆	☆	–	8

Rank by the beginning letter of the first authors

^a^1: Is the case definition adequate? 2: Representativeness of the cases. 3: Selection of Controls. 4: Definition of Controls

^b^Comparability: Comparability of cases and controls on the basis of the design or analysis. A maximum of two stars can be given for Comparability

^c^A: Ascertainment of exposure. B: Same method of ascertainment for cases and controls. C: Nonresponse rate

### Meta-analysis of gut microbiota

The included studies analyzed different taxa of gut microbiota in NAFLD patients at different levels. Based on available data, this meta-analysis primarily focused on alterations at the genus level, including *Escherichia*, *Prevotella*, *Streptococcus*, *Coprococcus*, *Faecalibacterium*, *Ruminococcus*, *Bacteroides*, *Bifidobacterium*, *Blautia*, *Clostridium*, *Dorea*, *Lactobacillus*, *Parabacteroides* and *Roseburia*.

**Fig. 2:**
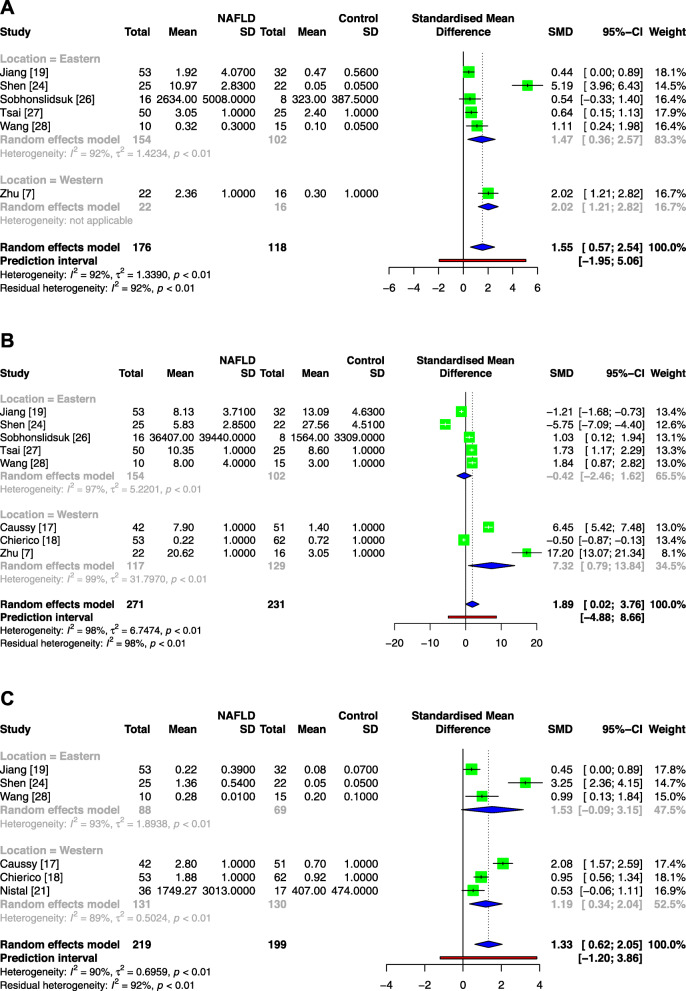
Meta-analysis comparing the abundance of *Escherichia* (**a**), *Prevotella* (**b**) and *Streptococcus* (**c**) between NAFLD patients and healthy controls. Abbreviations: NAFLD, nonalcoholic fatty liver disease; Tau^2^, tau-squared; Chi^2^, chi-square test; df, degrees of freedom; *I*^2^, I-squared

As noted in Fig. 2, the total SMDs of the genera *Escherichia*, *Prevotella* and *Streptococcus* were 1.55 [95% CI: 0.57, 2.54], 1.89 [95% CI: 0.02, 3.76] and 1.33 [95% CI: 0.62, 2.05], respectively, revealing that all these genera exhibited increased abundance in NAFLD. Significant heterogeneities were noted in the included studies of the three genera with *I*^2^ values greater than 50% and *p* values less than 0.1. In the subgroup analysis, the results of the two subgroups of the genus *Escherichia* were consistent with the overall results, while in the analysis of the genera *Prevotella* and *Streptococcus*, the results of the Eastern subgroups were not significantly different.

**Fig. 3:**
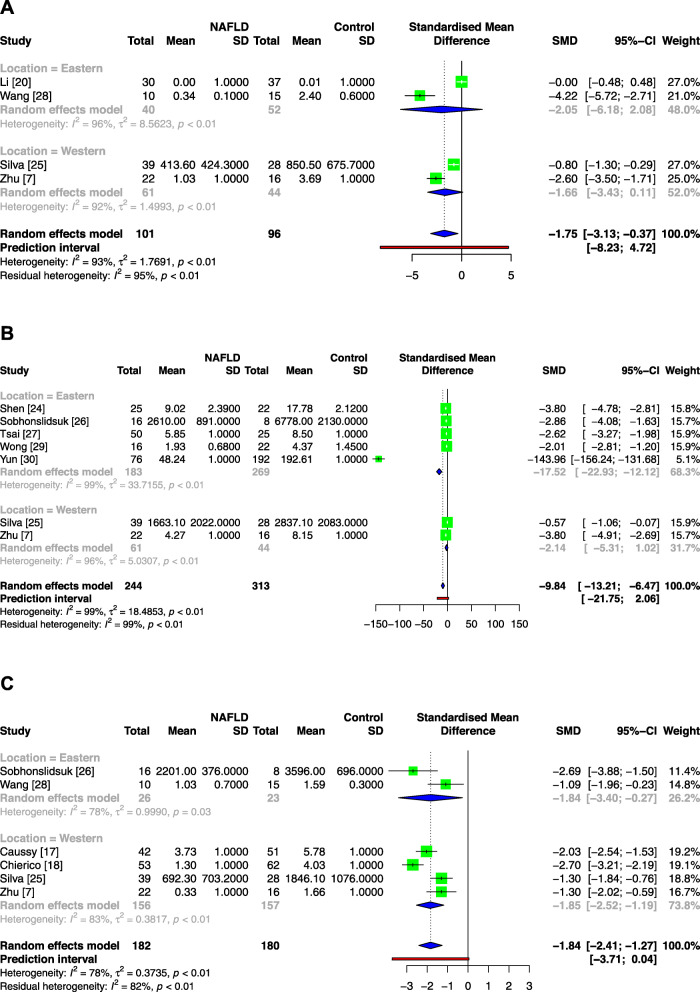
Meta-analysis comparing the abundance of *Coprococcus* (**a**), *Faecalibacterium* (**b**) and *Ruminococcus* (**c**) between NAFLD patients and healthy controls. Abbreviations: NAFLD, nonalcoholic fatty liver disease; Tau^2^, tau-squared; Chi^2^, chi-square test; df, degrees of freedom; *I*^2^, I-squared

In Fig. 3, the total SMDs of the genera *Coprococcus*, *Faecalibacterium* and *Ruminococcus* were − 1.75 [95% CI: − 3.13, − 0.37], − 13.23 [95% CI: − 17.59, − 8.87] and − 1.84 [95% CI: − 2.41, − 1.27], respectively, revealing that these genera were deficient in NAFLD patients. Significant heterogeneities were also noted. In the subgroup analysis, neither subgroup of the genus *Coprococcus* showed significant differences, the Western subgroup of the genus *Faecalibacterium* did not reach a significant difference either, and two subgroups of the genus *Ruminococcus* were consistent with the overall results.

**Fig. 4:**
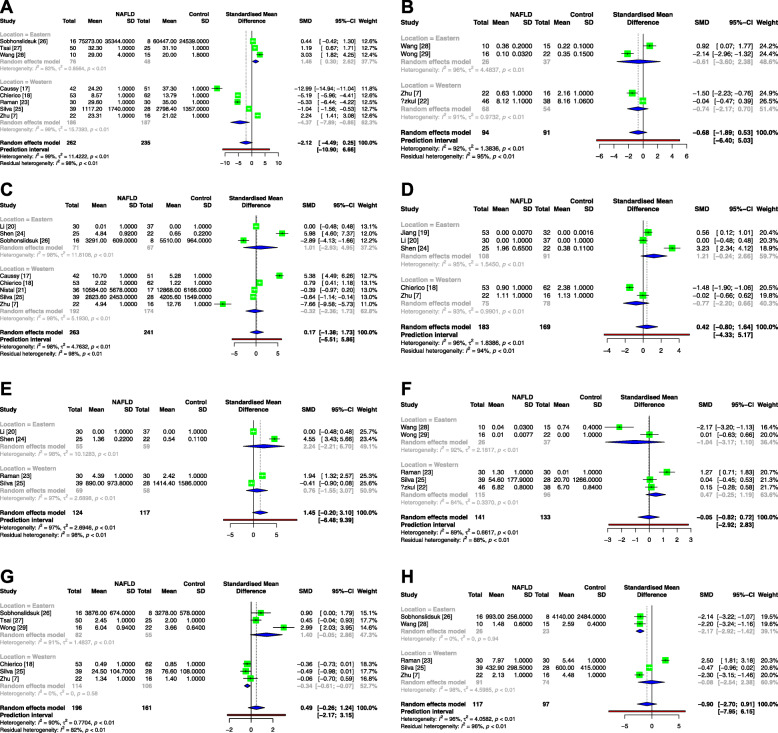
Meta-analysis comparing the abundance of *Bacteroides* (**a**), *Bifidobacterium* (**b**), *Blautia* (**c**), *Clostridium* (**d**), *Dorea* (**e**), *Lactobacillus* (**f**), *Parabacteroides* (**g**) and *Roseburia* (**h**) between NAFLD patients and healthy controls. Abbreviations: NAFLD, nonalcoholic fatty liver disease; Tau^2^, tau-squared; Chi^2^, chi-square test; df, degrees of freedom; *I*^2^, I-square

Regarding the genera *Bacteroides*, *Bifidobacterium*, *Blautia*, *Clostridium*, *Dorea*, *Lactobacillus*, *Parabacteroides* and *Roseburia* (Fig. 4), no significant differences were observed between the NAFLD patients and healthy controls in the overall analysis. Interestingly, in the subgroup analysis, the abundance of the genus *Bacteroides* in the NAFLD patients was decreased in the Western population but increased in the Eastern population. Moreover, the genera *Parabacteroides* and *Roseburia* in the NAFLD patients presented decreased abundance in the Western and Eastern subgroups, respectively.

### Analysis of publication bias

Supplemental Figure 1 and Supplemental Table 1 provide an analysis of the funnel plots and Egger’s and Begg’s tests for publication bias. According to Egger’s and Begg’s tests, none of the analyses presented publication bias.

### Sensitivity analysis

The sensitivity analysis (Supplemental Figure 2) showed that omitting each single study did not significantly change the results in the analysis for the genera *Escherichia, Streptococcus, Faecalibacterium* and *Ruminococcus.* However, after removing some of the included studies, the results of the genera *Prevotella* and *Coprococcus* did not reach significant differences. The results of the credibility ceiling test for ceilings of 0–40% are shown in Supplemental Table 2. All 3 meta-analyses that retained statistical significance with c = 20% or 40% for abundance changes of genera [*Coprococcus*, *Faecalibacterium*, *Ruminococcus*] in NAFLD. The meta-analysis on *Escherichia*, *Prevotella* and NAFLD none survived a 20% ceiling, whereas the *Coprococcus* remained statistically significant even with 20% ceiling but not 40% ceiling.

### Meta regression analysis of BMI and ALT for the significant genera

On univariate meta-regression model (Supplemental Figure 3), when plotting log odds ratio of mean different abundance of bacterial genus for NAFLD versus control patients (y-axis) against alanine aminotransferase (ALT) or body mass index (BMI) levels age (x-axis), significant association was found between BMI and *Faecalibacterium* (log regression coefficient β = 2.38, *P* = 0.00001), and *Prevotella* (regression coefficient β = 1.0554, *P* = 0.0005). While ALT levels was associated with different abundance of *Faecalibacterium* (log regression coefficient β = 1.11, *P* = 0.001), *Prevotella* (log regression coefficient β = 0.2018, *P* = 0.031), *Streptococcus* (log regression coefficient β = 0.09, *P* = 0.041). After multivariate adjustments, BMI remained significant for *Prevotella* (log regression coefficient β = 1.20, *P* = 0.019), while both BMI and ALT were significant for *Faecalibacterium* (log regression coefficient β = − 6.2, *P* = 0.00001 and β = 2.51, respectively, both *P* < 0.00001).

## Discussion

To our knowledge, this study is the first meta-analysis to investigate microbial shifts in NAFLD patients. This meta-analysis included 15 studies that assessed alterations in gut microbiota between 577 NAFLD patients and 688 healthy controls from eight different countries. Although the bacterial composition of the gut microbiota varies among individuals, the current study provides evidence for the existence of an overall profile of gut microbiota in NAFLD. Specifically, *Escherichia*, *Prevotella* and *Streptococcus* levels were increased in NAFLD patients (with 95% prediction interval of − 1.95 to 5.06, − 4.88 to 8.66 and − 1.2 to 3.86, respectively), while *Coprococcus*, *Faecalibacterium* and *Ruminococcus* were decreased (with 95% prediction interval of − 8.23 to 4.72, − 21.75 to 2.06 and − 3.71 to 0.04, respectively). However, no differences in the abundance of *Bacteroides*, *Bifidobacterium*, *Blautia*, *Clostridium*, *Dorea*, *Lactobacillus*, *Parabacteroides* or *Roseburia* were confirmed between the NAFLD patients and healthy controls.

Patients with NAFLD exhibited an increased proportion of gut *Enterobacter* (*Escherichia*) and *Streptococcus* in this study. Five studies [7, 19, 22, 24, 28] detected overgrowth of potentially antigenic bacteria, including *Enterobacter* (*Escherichia*) and *Streptococcus*, which is consistent with this study. Overgrowth of the *Proteobacteria* phylum (especially *Escherichia coli* and *Enterobacteriaceae* families) might increase intestinal permeability and portal LPS levels, which could trigger inflammasome activation and contribute to liver injury [31]. Moreover, *Escherichia coli* and other *Enterobacteriaceae* families produce ethanol [32], which might be responsible for the overproduction of endogenous ethanol that is involved in the development of NASH.

*Prevotella* has been identified as a fruit- and vegetable-rich diet-associated species that is also linked with short-chain fatty acid (SCFA) production [33]. The overall microbial differences across the studies revealed an increase in *Prevotella* in NAFLD. The reasons for the different conclusions across studies are not clear. One possible explanation might be that dietary habits, inflammatory conditions, and age confounded the relationship between *Prevotella* and NAFLD susceptibility [34]. Another probable explanation might be the different roles of the varied *Prevotella copri* strains in different genetic backgrounds [33]. Further studies analyzing *Prevotella* abundance at the strain level in NAFLD patients remain necessary.

It was also found that the abundance of *Coprococcus, Faecalibacterium and Ruminococcus* was reduced in NAFLD patients. *Ruminococcaceae* and *Faecalibacterium* have been well demonstrated to produce SCFAs via fermentation of dietary soluble fibers, which can activate their free fatty acid receptors (FFARs), including G-protein coupled receptor 43 and 41 (GPR43, GPR41) [35]. These pathways inhibit proinflammatory functions on neutrophils, monocytes and macrophages, thereby reducing the generation of tumor necrosis factor (TNF)-α and monocyte chemotactic protein-1 [36]. As SCFA-rich diets showed efficacy in alleviating insulin resistance and inflammation in both experimental mouse models and clinical trials [36], decreased numbers of *Ruminococcaceae* and *Faecalibacterium* may lower the SCFA levels in the gut, thereby escalating gut inflammation involved in the pathogens of NAFLD.

In this study, significant heterogeneities were present among the evaluated studies, which might be attributed to population characteristics, diet, obesity degree, NAFLD severity, associated comorbidities (i.e., metabolic syndrome), detection methods and other factors. Although subgroup analysis stratified with Eastern and Western to further demonstrated the potential effect of different regions with varied lifestyles, another important subgroup analysis of disease related features of NAFLD including steatosis degree, inflammation severity or fibrosis stages were not conducted due to the lack of these detailed information in the included studies.

### Study strength and limitations

This study is the first to identify microbial signatures in NAFLD patients via a meta-analysis. A subgroup analysis was also conducted by classifying the research locations to validate the generalities of the results in the overall generation. Certain limitations were observed in this meta-analysis. First, the included studies only analyzed gut microbiomes recovered in the stool; however, several studies have indicated that mucosa-associated bacteria might greatly differ from those recovered in stool and could play a more important role in the pathogenesis of associated diseases [31, 32]. In addition, most of the analyses presented significant heterogeneities, which can only be minimized but not eliminated using a random-effects model. Third, several eligible studies [37–41] were excluded because of lack of necessary data. Fourth, the sensitivity of Egger’s and Begg’s test is low in the context of small sample size (most of which does not exceed 100) of the included studies. Last but not least, meta-regression was performed for BMI and ALT values based on aggregate data; therefore, the risk of ecollogic fallacy exists.

## Conclusion

In conclusion, this study sought to assess gut microbial signatures in NAFLD patients via a meta-analysis. This study confirmed increases in the genera *Escherichia*, *Prevotella* and *Streptococcus* and decreases in the genera *Coprococcus*, *Faecalibacterium* and *Ruminococcus* as compositional patterns of fecal microbiota in NAFLD patients. Furthermore, BMI may contribute to the abundance change of *Faecalibacterium* and *Prevotella* in NAFLD relative to the control, whereas inflammation markers of ALT was associated with the abundance change of *Streptococcus* and *Faecalibacterium* in the meta regression analysis. This work will help identify specific differences in the proportion of several bacterial taxa in stool samples correlated with the presence of NAFLD, which will provide evidence for the design of probiotic and antibiotic treatments for NAFLD patients. This study suggested that targeting these gut microbiota in NAFLD patients may be another approach for treatment in the future.

## Supplementary Information

**Additional file 1.** Supplemental Material.

## Data Availability

All data generated or analysed during the current study are included in this published article and its supplementary information files.

## Abbreviations

CI: Confidence interval
FFARs: Free fatty acid receptors
GPR: G-protein coupled receptor
LPS: Lipopolysaccharide
MeSH: Medical subject heading
NAFL: Non-alcoholic fatty liver
NAFLD: Nonalcoholic fatty liver disease
NASH: Nonalcoholic steatohepatitis
NOS: Newcastle Ottawa scale
PRISMA: Preferred reporting items for systematic reviews and meta-analyses
SCFAs: Short-chain fatty acids
SD: Standard deviation
SMD: Standard mean difference
TNF: Tumor necrosis factor
